# Association of Mast Cell Activity With Chronic Gingivitis, Chronic Periodontitis, and Aggressive Periodontitis in Adults: A Histochemical Observational Study

**DOI:** 10.7759/cureus.86762

**Published:** 2025-06-25

**Authors:** Manisha Mallik, Anindita Banerjee, Dipankar Samaddar, Nitubroto Biswas, Toshi Toshi, Nikhil Raj, Seema Gupta

**Affiliations:** 1 Department of Periodontology, Buddha Institute of Dental Sciences and Hospital, Patna, IND; 2 Department of Oral Pathology, Update Dental Clinic, Kolkata, IND; 3 Department of Conservative Dentistry and Endodontics, Shree Ram Multispeciality Dental Clinic, Patna, IND; 4 Department of Orthodontics, Kothiwal Dental College and Research Centre, Moradabad, IND

**Keywords:** degranulation, gingiva, mast cell, periodontitis, toluidine blue

## Abstract

Introduction: Periodontal diseases, including chronic gingivitis, chronic periodontitis, and aggressive periodontitis, are driven by complex immune responses, with mast cells (MCs) playing a pivotal role through the release of mediators during degranulation. Although MC involvement in chronic periodontal conditions has been documented, its role in aggressive periodontitis and the relationship between MC degranulation and inflammation intensity remain poorly understood. This study aimed to quantify MCs and assess their degranulation in gingival tissues across healthy gingiva, chronic gingivitis, chronic periodontitis, and aggressive periodontitis using histochemical staining to elucidate their role in periodontal inflammation and disease progression.

Materials and methods: This cross-sectional study included 120 systemically healthy subjects recruited from the Periodontology Outpatient Department. The groups included healthy periodontium (n = 30), chronic gingivitis (n = 30), chronic periodontitis (n = 30), and aggressive periodontitis (n = 30) groups. Gingival biopsies were obtained, fixed, sectioned, and stained with toluidine blue to identify intact and degranulated MCs in juxta-epithelial (Z1) and deep connective tissue (Z2) zones. MC counts were performed under 40x magnification, with examiner calibration to ensure reliability (intraclass correlation (ICC) ≥ 0.85). Nonparametric tests (Kruskal-Wallis with Dunn’s test) were used to analyze MC density and degranulation, with a significance level of p < 0.05.

Results: MC counts were highest in chronic periodontitis (7.10 ± 2.50 in Z1, 6.70 ± 2.10 in Z2) and aggressive periodontitis (6.10 ± 1.70 in Z1, 6.50 ± 2.00 in Z2), followed by chronic gingivitis and healthy gingiva (p < 0.00001). Degranulated MCs were significantly elevated in diseased groups, particularly chronic periodontitis (4.69 ± 2.09 in Z1, 4.51 ± 1.89 in Z2) compared to healthy controls (p = 0.0001). Intact MCs showed less variation, with significant differences between the healthy and aggressive periodontitis groups (p = 0.045).

Conclusion: Increased MC degranulation was associated with periodontal disease severity, particularly in chronic and aggressive periodontitis, suggesting a key role of MC degranulation in inflammatory progression. These findings highlight the potential of MCs as therapeutic targets in periodontal management.

## Introduction

Periodontal diseases, including gingivitis and periodontitis, are chronic pathologies primarily driven by bacterial etiologies, with the host’s immunoinflammatory response to microbial plaques playing a pivotal role in their pathogenesis [1]. Although dental plaque, a complex biofilm of microorganisms, serves as the initiating factor, it is insufficient to cause significant periodontal tissue destruction [2]. Instead, the progression of these diseases results from a dynamic interplay between the microbial biofilm and the host’s immune-inflammatory response, with the latter contributing to approximately 80% of the risk of periodontal tissue damage [1,2]. This intricate relationship underscores the importance of understanding the cellular and molecular mechanisms that govern periodontal health and disease [2].

Central to the immunoinflammatory response in periodontal tissues is the infiltration of various immune cells, among which mast cells (MCs) have emerged as a significant player [3]. MCs are enigmatic immune cells that continue to intrigue researchers owing to their multifaceted roles in health and disease [3]. These cells are predominantly located in tissues interfacing with the external environment, such as the skin, respiratory mucosa, and gastrointestinal tract, and are positioned as early responders to invading pathogens [4]. In the oral cavity, MCs are ubiquitous across all tissues, including the gingiva, suggesting a critical role in maintaining gingival homeostasis and potentially influencing the progression of periodontal diseases, as well as tissue healing [5].

Histological examinations of inflamed periodontal tissues reveal a high density of MCs, often equal to or surpassing the number of macrophages in lesions adjacent to periodontal pockets [3,4]. These observations highlight their potential roles in periopathogenesis. MCs are equipped with granules containing potent mediators such as histamine, cytokines, and proteases, which are released upon degranulation in response to microbial or immune stimuli [5]. Degranulation can amplify inflammatory responses, modulate immune cell recruitment, and influence tissue remodelling, all of which are critical processes in periodontal disease progression [6]. However, the precise role of MCs in periodontal diseases, particularly their density, distribution, and functional contributions to different disease states, remains a subject of ongoing debate and investigation.

While the presence of MCs in chronic periodontitis and gingivitis has been documented, there is a notable paucity of research exploring their role in aggressive periodontitis, a severe and rapidly progressing form of the disease [6]. Furthermore, the relationship between MC degranulation and the intensity of periodontal inflammation has not been fully elucidated. To address these gaps, this study aimed to quantify MCs in gingival tissues across a spectrum of periodontal conditions, such as healthy gingiva, chronic gingivitis, chronic periodontitis, and aggressive periodontitis, using histochemical staining techniques. By comparing MC densities and analyzing the extent of degranulation, this study sought to elucidate the role of MCs in periodontal inflammation and their potential contribution to disease progression and tissue repair. Understanding these mechanisms might offer new insights into the immunopathology of periodontal diseases and inform targeted therapeutic strategies.

## Materials and methods

Study design and setting

This cross-sectional, observational, prospective study was conducted in the Department of Periodontology, Buddha Institute of Dental Sciences and Hospital, Patna, India, from April 2022 to May 2023. The study adhered to the ethical standards outlined in the Declaration of Helsinki and was approved by the Institutional Ethical Committee (93/BIDSH). This investigation focused on quantifying MCs in gingival tissues across four periodontal conditions: healthy periodontium, chronic gingivitis, chronic periodontitis, and aggressive periodontitis. Written informed consent was obtained from all the patients after explaining the study to them in their native language.

Sample size calculation

The sample size was calculated using G*Power software (version 3.1.9.7, Heinrich-Heine-Universität Düsseldorf, Düsseldorf, Germany) based on previous studies reporting MC density in periodontal tissues. Assuming an effect size of 0.5, a power of 80%, and an alpha error of 0.05, a minimum of 27 patients per group was required. To account for potential histological processing losses, 30 patients were recruited for each group, totalling 120 patients.

Subject selection

Patients were recruited from the Outpatient Department (OPD). The inclusion criteria were as follows: systemically healthy patients aged 18-50 years with a minimum of 20 natural teeth were included. Group I (healthy periodontium) comprised patients with probing depth (PD) ≤ 3 mm, no bleeding on probing (BOP), and a Gingival Index (GI) score of 0 [7]. Group II (chronic gingivitis) included patients with PD ≤ 3 mm, GI ≥ 1, and presence of BOP without clinical attachment loss (CAL). Group III (chronic periodontitis) consisted of patients with PD and CAL ≥ 5 mm at ≥ 30% of sites, GI ≥ 2, and radiographic evidence of horizontal bone loss. Group IV (aggressive periodontitis) included systemically healthy patients presenting with rapid attachment and bone loss, CAL ≥ 5 mm and PD ≥ 6 mm at ≥ 3 permanent teeth, including at least one incisor and one first molar, minimal microbial deposits inconsistent with destruction severity, and radiographic evidence of vertical bone defects. 

Exclusion criteria encompassed patients with a history of tobacco or alcohol use, antibiotic use, or use of anti-inflammatory drugs within the past three months, or steroid therapy were excluded. Medically compromised patients, patients with systemic diseases affecting the periodontium (such as diabetes, immunosuppression), pregnant or lactating females, and sites with endodontic lesions or suppuration were also excluded to minimize confounding factors. Additional exclusion criteria included having received periodontal therapy or professional cleaning within the last six months, and the presence of fixed orthodontic appliances or prostheses that could influence periodontal health.

Methodology

In Group I, gingival biopsies were obtained from patients undergoing esthetic crown lengthening or orthodontic extractions. For Groups II, III, and IV, the sites with the highest inflammation or greatest attachment loss were selected. Local anesthesia was administered using 2.0% lignocaine hydrochloride (Lox 2%, Neon Laboratories Ltd., Mumbai, India) via infiltration. Tissue samples were excised using a No. 15 Bard-Parker blade (Kehr Surgical Pvt. Ltd., Kanpur, India) before any periodontal intervention to ensure baseline tissue characteristics.

The biopsy specimens were immediately fixed in 10% neutral buffered formalin (Sigma-Aldrich, St. Louis, MO, USA) for 24 hours. Tissues were dehydrated through a graded ethanol series (70%, 95%, and 100%; Merck KGaA, Darmstadt, Germany), cleared in xylene (Merck KGaA, Darmstadt, Germany), and embedded in paraffin wax (Leica Biosystems, Wetzlar, Germany). Sections of 5 μm thickness were prepared using a rotary microtome (Leica RM2245, Leica Biosystems, Wetzlar, Germany).

**Figure 1:**
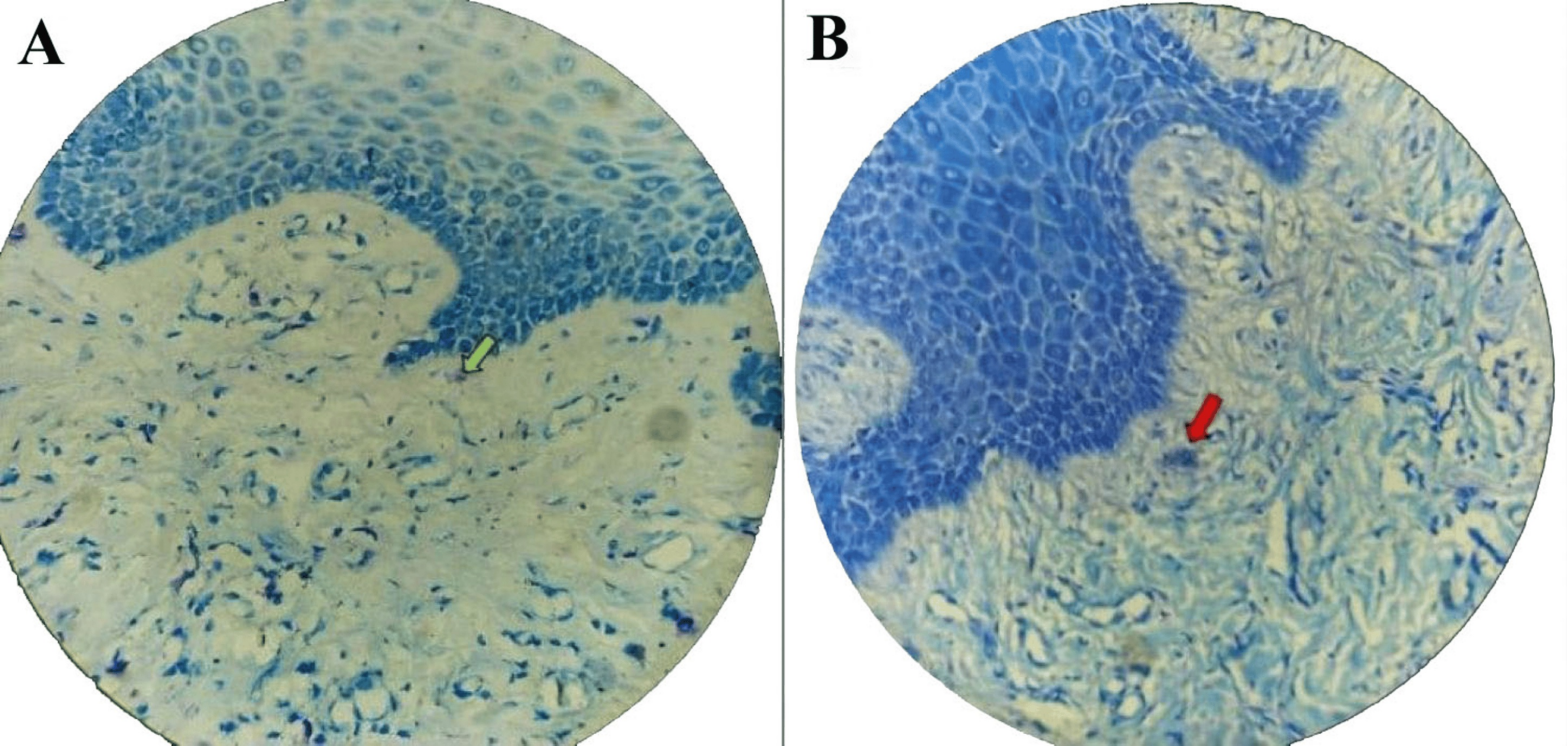
Toluidine blue stained section of gingival tissue at 40x magnification (A) intact mast cells (green arrow) and (B) degranulated mast cells (red arrow) This figure depicts histological sections of gingival tissue of patients included in this study

The sections were deparaffinized in xylene and rehydrated using descending concentrations of ethanol in distilled water. Staining was performed with 1% toluidine blue (Sigma-Aldrich, St. Louis, MO, USA) for 30 minutes, followed by rinsing in phosphate-buffered saline (PBS, pH 7.4; Thermo Fisher Scientific, Waltham, MA, USA). Sections were rapidly dehydrated using 70%, 96%, and absolute ethanol (Merck KGaA, Darmstadt, Germany), cleared in xylene, mounted with DPX mountant (Merck KGaA, Darmstadt, Germany), and cover-slipped for microscopic analysis (Figure 1).

MCs were identified using toluidine blue staining, which induces metachromasia by interacting with MC granules. Two morphological stages were recognized: intact MCs, characterized by dark blue/purple staining, intense metachromasia, dense granules, and an intact cell membrane, and degranulated MCs, characterized by reduced metachromasia, extruded metachromatic granules, and discontinuous cell membranes. MCs were quantified in two zones: zone 1 (juxta-epithelial region) and zone 2 (deep connective tissue).

MC counts (intact and degranulated) were determined by examining three consecutive high-power fields (HPF, 40x magnification) in each zone using a binocular scientific microscope (BX53; Olympus Corporation, Tokyo, Japan), ensuring no field overlap. The average number of MCs per HPF was calculated separately for zones 1 and 2. Images were captured using a digital camera (DP73; Olympus Corporation, Tokyo, Japan).

Two independent examiners, trained in histopathology, performed MC counting. Calibration was conducted on 10% of samples prior to the study, achieving an intraclass correlation coefficient (ICC) of ≥ 0.85 for both intact and degranulated MC counts. Discrepancies were resolved through consensus. A single examiner performed final counts to ensure consistency, with 10% of the slides re-evaluated after two weeks to confirm intra-examiner reliability (ICC ≥ 0.90).

Statistical analysis

Data were entered into MS Excel (Microsoft Corporation, Redmond, Washington, United States) and analyzed using GraphPad Prism (version 6.0, GraphPad Software, San Diego, CA, USA). Descriptive statistics (mean and standard deviation) were calculated for MC counts. The Shapiro-Wilk test confirmed nonnormal data distribution, prompting the use of nonparametric tests. The Kruskal-Wallis test, followed by Dunn’s multiple comparison test, was used to compare MC counts across all four groups. Statistical significance was set at p < 0.05.

## Results

Sex distribution was relatively balanced across all the groups. The healthy gingiva group had a slightly higher proportion of females, whereas the chronic and aggressive periodontitis groups showed a marginal male predominance. The mean age was lowest in the healthy gingiva group (32.45 ± 8.21 years) and highest in the aggressive periodontitis group (38.56 ± 9.12 years), suggesting a potential association between advancing age and severe periodontal disease. Chronic gingivitis and periodontitis groups exhibited intermediate age ranges (36.78 ± 6.34 and 34.55 ± 8.34 years, respectively), though no strict linear trend was observed (Table 1). 

**Table 1:** Descriptive analysis of study population Data for sex presented in frequency (n) and percentage (%) and data for age in mean and standard deviation (SD)

Groups	Male	Female	Age (years)
	n (%)	n (%)	Mean ± SD
Healthy gingiva	12 (10%)	18 (15%)	32.45 ± 8.21
Chronic gingivitis	14 (12%)	16 (13%)	36.78 ± 6.34
Chronic periodontitis	17 (14%)	13 (11%)	34.55 ± 8.34
Aggressive periodontitis	14 (12%)	16 (13%)	38.56 ± 9.12

The mean MC counts in Z1 were highest in chronic periodontitis (7.10 ± 2.50), followed by aggressive periodontitis (6.10 ± 1.70), healthy gingiva (4.74 ± 2.07), and chronic gingivitis (4.73 ± 1.12). In Z2, chronic periodontitis showed the highest count (6.70 ± 2.10), followed by aggressive periodontitis (6.50 ± 2.00), chronic gingivitis (5.24 ± 1.09), and healthy gingiva (4.29 ± 1.55). The Kruskal-Wallis test revealed statistically significant differences in MC distribution among the groups for both tissue types (p < 0.00001). This suggested that MC infiltration increased significantly with periodontal disease severity, particularly in chronic periodontitis, indicating their role in inflammatory progression (Table 2).

**Table 2:** Comparison of average number of mast cells per high power field in juxta-epithelial (Z1) and deep connective tissue (Z2) regions among the study groups Data presented in mean and standard deviation (SD); *p-value < 0.05: significant using Kruskal-Wallis test

Groups	N	Mast cells (Z1)	Mast cells (Z2)
		Mean ± SD	Mean ± SD
Healthy gingiva	30	4.74 ± 2.07	4.29 ± 1.55
Chronic gingivitis	30	4.73 ± 1.12	5.24 ± 1.09
Chronic periodontitis	30	7.10 ± 2.50	6.70 ± 2.10
Aggressive periodontitis	30	6.10 ± 1.70	6.50 ± 2.00
p-value		<0.00001*	<0.00001*

Pairwise comparisons using Dunn’s test revealed significant differences in MC counts between the groups. At Z1, healthy gingiva showed significant differences compared to chronic periodontitis (p = 0.0002) and aggressive periodontitis (p = 0.007), but not with chronic gingivitis (p = 0.981). Chronic gingivitis differed significantly from both chronic gingivitis (p = 0.0001) and aggressive periodontitis (p = 0.0005). No differences were observed between the chronic and aggressive periodontitis groups (p = 0.071). For connective tissue, all comparisons were significant (p < 0.05), except for chronic versus aggressive periodontitis (p = 0.707). These findings suggested that MC infiltration varied significantly with disease severity, particularly distinguishing periodontitis from gingivitis and healthy tissue (Table 3).

**Table 3:** Pairwise comparison of average number of mast cells per high power field in juxta-epithelial (Z1) and deep connective tissue (Z2) regions using Dunn’s test *p-value < 0.05: significant using Dunn’s test

Paired groups		Mast cells (Z1)		Mast cells (Z2)	
		t value	p-value	t	p-value
Healthy gingiva	Chronic gingivitis	0.02	0.981	2.74	0.008*
Healthy gingiva	Chronic periodontitis	3.98	0.0002*	5.05	0.0001*
Healthy gingiva	Aggressive periodontitis	2.78	0.007*	4.78	0.0001*
Chronic gingivitis	Chronic periodontitis	4.73	0.0001*	3.37	0.001*
Chronic gingivitis	Aggressive periodontitis	3.68	0.0005*	3.02	0.003*
Chronic periodontitis	Aggressive periodontitis	1.81	0.071	0.37	0.707

This study revealed significant variations in the MCs across different periodontal conditions. In Z1, intact MCs were highest in healthy gingiva (3.11 ± 1.67) and lowest in aggressive periodontitis (2.12 ± 0.69), with statistically significant differences among groups (p = 0.0301). Conversely, Z1 degranulated MCs showed marked elevation in chronic periodontitis (4.69 ± 2.09) compared to healthy gingiva (1.63 ± 0.74), with highly significant intergroup differences (p = 0.0001). In Z2, intact MCs did not differ significantly (p = 0.2106), while degranulated forms again demonstrated a substantial increase in diseased groups (chronic periodontitis: 4.51 ± 1.89; aggressive periodontitis: 4.37 ± 1.81) versus healthy controls (1.61 ± 0.63) (p = 0.0001). These findings indicated that periodontal disease progression was associated with significant MC degranulation, rather than changes in intact cell numbers, suggesting an important role for MC activation in periodontal inflammation. The similar patterns between chronic and aggressive periodontitis implied comparable inflammatory mechanisms despite differing disease courses (Table 4).

**Table 4:** Comparison of average number of intact and degranulated mast cells per high power field in juxta-epithelial (Z1) and deep connective tissue (Z2) regions among the four groups Data presented in mean ± standard deviation; *p-value < 0.05: significant using Kruskal-Wallis test

Mast cells		Healthy gingiva (mean ± SD)	Chronic gingivitis (mean ± SD)	Chronic periodontitis (mean ± SD)	Aggressive periodontitis (mean ± SD)	p-value
Mast cells (Z1)	Intact	3.11 ± 1.67	2.14 ± 0.60	2.37 ± 0.87	2.12 ± 0.69	0.0301*
	Degranulated	1.63 ± 0.74	2.59 ± 0.82	4.69 ± 2.09	3.97 ± 1.79	0.0001*
Mast cells (Z2)	Intact	2.68 ± 1.12	2.29 ± 0.60	2.18 ± 1.13	2.17 ± 0.78	0.2106
	Degranulated	1.61 ± 0.63	2.96 ± 0.80	4.51 ± 1.89	4.37 ± 1.81	0.0001*

Pairwise comparisons revealed significant differences in MC characteristics between the periodontal statuses. For Z1 intact MCs, healthy gingiva showed significantly higher counts than chronic gingivitis (p = 0.004), chronic periodontitis (p = 0.035), and aggressive periodontitis (p = 0.004). However, no significant differences were observed between the disease groups (P > 0.05). Z1 degranulated MCs demonstrated highly significant increases in all disease groups compared to healthy gingiva (p ≤ 0.003), with chronic gingivitis differing from both forms of periodontitis (p ≤ 0.003). In Z2, degranulated MCs showed similar patterns (p ≤ 0.003 for all disease versus healthy comparisons), whereas intact MCs only differed significantly between healthy gingiva and aggressive periodontitis (p = 0.045). Notably, neither intact nor degranulated forms differed between chronic and aggressive periodontitis in any compartment (p > 0.05), suggesting similar MC responses in these disease states, despite their clinical differences. The consistent elevation of degranulated MCs across all disease groups versus healthy tissue underscored their potential role in the pathogenesis of periodontal inflammation (Table 5).

**Table 5:** Pairwise comparison of average number of intact and degranulated mast cells per high power field in juxta-epithelial (Z1) and deep connective tissue (Z2) regions *p-value < 0.05: significant using Dunn’s test

Paired groups		Mast cells (Z1)				Mast cells (Z2)			
		Intact		Degranulated		Intact		Degranulated	
		t value	p-value	t value	p-value	t value	p-value	t value	p-value
Healthy gingiva	Chronic gingivitis	2.99	0.004*	4.76	0.001*	1.68	0.098	7.26	0.001*
Healthy gingiva	Chronic periodontitis	2.15	0.035*	7.55	0.001*	1.72	0.091	7.97	0.001*
Healthy gingiva	Aggressive periodontitis	3.01	0.004*	6.61	0.001*	2.04	0.045*	7.88	0.001*
Chronic gingivitis	Chronic periodontitis	1.19	0.238	5.12	0.001*	0.47	0.639	4.13	0.001*
Chronic gingivitis	Aggressive periodontitis	0.11	0.905	3.83	0.003*	0.66	0.506	3.90	0.003*
Chronic periodontitis	Aggressive periodontitis	1.13	0.222	1.42	0.157	0.03	0.968	0.29	0.770

## Discussion

The present study aimed to quantify and qualitatively assess MCs in periodontal tissues across four distinct groups: healthy periodontium, chronic gingivitis, chronic periodontitis, and aggressive periodontitis. These findings provided significant insights into the role of MCs in periodontal health and disease, particularly their density, distribution, and degranulation patterns in Z1 and Z2 regions. These results contribute to a growing body of evidence suggesting that MC are pivotal in the immunoinflammatory response underlying periodontal diseases, with implications for both disease progression and potential therapeutic strategies [3].

The study found that the highest number of MCs per HPF was observed in the chronic periodontitis group, closely followed by that in the aggressive periodontitis group. This observation aligns with previous research indicating that MC density increases in inflamed periodontal tissues compared with healthy tissues [3,8]. The significantly lower MC counts in the healthy periodontium compared to both periodontitis groups underscore the association between MC infiltration and the inflammatory state of the tissue. However, a study by Robinson and De Marco [9] reported a reduction in the number of MCs with increasing inflammation. This disparity in results could have been due to the fact that subtly discolored degranulated MCs might have contributed to an inaccurate assessment of their population. Additionally, variations in the methodologies and timing employed for sample collection and staining procedures have elucidated this substantial variance.

The results of our study indicated a lack of significant differences in MC counts between patients with chronic periodontitis and those with aggressive periodontitis. Although chronic and aggressive periodontitis differed in their clinical presentation, microbial profile, and progression rate, the similarity in MC density suggested that MCs might play a comparable role in the immunoinflammatory cascade in both conditions. This finding challenges the notion that aggressive periodontitis, characterized by rapid tissue destruction despite minimal microbial deposits, might involve distinct immune cell dynamics compared with chronic periodontitis. Instead, it supports the hypothesis that MCs are central to the host response to periodontal diseases, regardless of the specific clinical phenotype.

Although no significant differences were observed in the number of intact and degranulated MCs between chronic periodontitis and aggressive periodontitis groups, the number of MCs was higher in the chronic periodontitis group. This finding is in agreement with those of previous studies by Fattahi et al. [10] and Vahabi et al. [11]. Vahabi et al. [11] examined the correlation between MC quantity and chronic periodontitis. The findings of this research indicated that the MC quantities in the cohort suffering from chronic periodontitis were elevated compared to those in the group with invasive periodontitis and the healthy control group. Moreover, individuals diagnosed with acute periodontitis did not demonstrate increased MC counts compared to healthy individuals. An alternative rationale for the elevated presence of MC in cases of moderate to severe periodontitis may be associated with the diverse functions of these cells. In the context of periodontal diseases, MC serves not only as a mediator of inflammation but also plays a pivotal role in angiogenesis. Consequently, an augmentation in their population could represent a fundamental aspect of the advancement of inflammatory alterations within the gingival tissue.

MCs contain granules that harbor preformed inflammatory mediators and can synthesize additional mediators upon stimulation [3]. Each MC contains 80-300 granules [3]. These granules comprise mucopolysaccharides, which bestow the metachromatic staining characteristic of MCs. Intact MCs exhibit a dense staining pattern, whereas granular leukocytes with compromised cell membranes appear lightly stained and are classified as degranulated. The functional status of MCs is indicated by their degranulation in response to various stimuli, which underpins their biological activity [12]. Upon activation, MCs may either experience explosive degranulation followed by granule resynthesis or release individual granules continuously into their surroundings, a phenomenon referred to as "piecemeal degranulation" [13].

MC can be visualized by Giemsa staining and various immunohistochemical techniques [14]. Nevertheless, the present study employed toluidine blue because of its ease of application and widespread availability. Toluidine blue is a cationic metachromatic dye that preferentially adheres to acidic constituents within the tissues [15]. The granules that house histamine and heparin avidly absorb this dye, resulting in a pronounced purple hue that facilitates the identification of MC.

Notably, the chronic gingivitis group exhibited a significantly higher MC count in the Z2 group than in the control group, but this difference was less pronounced in the Z1 group. This suggests a gradient of MC involvement that may correspond to the severity and depth of inflammation, with deeper tissues potentially reflecting a more chronic or sustained inflammatory response. Ramalingam et al. [16] hypothesized that the distribution of MCs across various levels of connective tissue signifies their involvement in distinct phases of the oral lichen planus. This assertion may be equally applicable to periodontal disease. In the current study, the two tissue zones, specifically Z1 and Z2, exhibited a heterogeneous distribution of MCs. The initial phase may entail dilation of blood vessels, facilitating the extravasation of lymphocytes. Subsequently, these lymphocytes are drawn toward the subepithelial zone [16]. Moreover, MCs may release various cytokines that contribute to the degradation of the extracellular matrix and promote the recruitment of lymphocytes to the juxta-epithelial regions [16].

The distribution of MC across the Z1 and Z2 regions also provided insight into their functional roles. The juxta-epithelial region, which is closer to the periodontal pocket and microbial biofilm, is likely the initial site of interaction between MC and invading pathogens [6]. The higher MC density in this region in diseased states might reflect their role as sentinel cells responding rapidly to microbial challenges. In contrast, Z2 may represent a reservoir for MCs that contributes to sustained inflammatory responses, as evidenced by the significant increase in MCs in chronic gingivitis compared to the control in this region [17]. These findings suggest that MC distribution is not uniform and may be influenced by proximity to the inflammatory stimulus and the chronicity of the disease.

Qualitative analysis of MCs, focusing on intact versus degranulated states, revealed important patterns related to disease progression. Intact MCs, characterized by intense metachromasia and dense granules, were most abundant in the healthy periodontium. This suggests that MCs remain largely quiescent in healthy tissues, potentially serving a homeostatic role in gingival tissue maintenance [17,18].

Degranulated MCs, identified by less intense metachromasia and extruded granules, were significantly more prevalent in diseased states than in healthy tissues. This increase in degranulation with disease progression from health to gingivitis and periodontitis aligns with the known role of MCs in amplifying inflammatory responses through the release of mediators such as histamine, cytokines, and proteases [12]. These mediators can enhance vascular permeability, recruit other immune cells, and contribute to tissue destruction, all of which are hallmarks of periodontal disease [3]. The significant increase in degranulated MCs in both chronic and aggressive periodontitis compared to chronic gingivitis further suggested that MC activation increased with the severity of periodontal inflammation.

The expression of matrix metalloproteinases (MMPs) 1, 2, and 8 is most pronounced in MC. MMPs play an essential role in the degradation of the principal constituents within the extracellular matrix [19]. Furthermore, tryptase possesses the ability to cleave the third component of collagen and activate latent collagenase, which may contribute to tissue destruction in periodontitis [20]. The transition from gingivitis to periodontitis signifies a transformation from a primarily T cell-mediated lesion to one predominantly characterized by B cell and plasma cell involvement. MCs can present antigens to T lymphocytes. The subsequent activation of T cells would, in turn, stimulate MCs, resulting in both degranulation and cytokine secretion [6,8,11].

Interestingly, this study found no significant differences in the degranulation patterns of MCs between chronic and aggressive periodontitis. This finding is noteworthy, given the distinct clinical and microbiological characteristics of these conditions. Aggressive periodontitis is often associated with specific pathogens, such as *Aggregatibacter actinomycetemcomitans*, and a more rapid rate of tissue destruction; however, the similarity in MC degranulation suggests a common pathway of immune activation [10,11,21]. This could imply that MCs respond to a broad range of inflammatory signals, regardless of the specific microbial trigger or disease kinetics. Alternatively, it may reflect a saturation point in MC activation where the extent of degranulation reaches a plateau in advanced disease states.

Clinical implications of the study

The findings of this study underscore the critical role of MCs in the inflammatory response in periodontal disease. MCs are strategically positioned at interfaces with the external environment, such as the gingival mucosa, making them among the first immune cells to encounter periodontal pathogens [6]. Their ability to release a wide array of mediators upon degranulation positions them as key orchestrators of the inflammatory cascade, bridging the innate and adaptive immune responses. The increased MC density and degranulation in diseased states suggest that they contribute to the chronicity and severity of periodontal inflammation, potentially exacerbating tissue damage through the release of destructive mediators. The similarity in MC behavior between chronic and aggressive periodontitis raises important questions regarding the specificity of immune responses in these conditions. While aggressive periodontitis is often considered a distinct entity owing to its rapid progression and genetic predispositions, the comparable MC profiles suggest that the host response, particularly at the level of MCs, may be a unifying feature. This could have implications for therapeutic strategies, as targeting MC activity (through antihistamines or MC stabilizers) might offer benefits across both forms of periodontitis.

Limitations and future directions

Although this study provides valuable insights, several limitations should be acknowledged. First, the sample size, although adequate for statistical analysis, should be expanded in future studies to enhance the generalizability of the findings. Second, toluidine blue staining, which is effective for identifying MCs, might not capture the full spectrum of MC phenotypes or functional states. Advanced techniques such as immunohistochemistry or flow cytometry can provide more detailed information about MC subpopulations and their specific mediators. Third, the study focused on two tissue zones (Z1 and Z2), but further granularity in analyzing the MC distribution (such as within specific gingival layers or in relation to blood vessels) could elucidate their spatial dynamics.

Future research should explore the molecular mechanisms underlying MC activation in periodontal disease. For example, identifying specific microbial- or host-derived signals that trigger MC degranulation could inform targeted interventions. Additionally, longitudinal studies examining MC dynamics over the course of periodontal disease progression and treatment could clarify their roles in disease resolution and tissue repair. Given the potential homeostatic role of MCs in healthy gingiva [8], investigations into their reparative functions, such as promoting angiogenesis or fibroblast activity, could uncover novel therapeutic applications.

## Conclusions

This study demonstrated that MCs play a significant role in periodontal diseases, with increased density and degranulation observed in chronic gingivitis, chronic periodontitis, and aggressive periodontitis compared to healthy periodontium. The highest MC counts were found in chronic periodontitis, closely followed by aggressive periodontitis, with no significant difference between the two, suggesting a shared immunoinflammatory role. Degranulated MCs were significantly more prevalent in diseased states, particularly periodontitis, indicating heightened MC activity with disease progression. These findings underscore the importance of MCs in the pathogenesis of periodontal diseases and suggest that targeting MC activity could be a potential therapeutic strategy for managing periodontal inflammation.
